# Management of prolonged post-operative pelvic pain after transurethral prostate surgery: a clinical real-world survey and international comparison of therapy regimens

**DOI:** 10.1186/s12894-025-01943-z

**Published:** 2025-09-17

**Authors:** Max Traeger, Tabea Walther, Jeremy Yuen-Chun Teoh, Marcelo Langer Wroclawski, Thomas Herrmann, Arkadiusz Miernik, Konrad Wilhelm, Maximilian Glienke, Philippe-Fabian Pohlmann, Christian Gratzke, Dominik Schoeb

**Affiliations:** 1 Department of Urology, Faculty of Medicine, Medical Centre, University of Freiburg, Hugstetter Strasse 55, Freiburg im Breisgau, 79106 Germany; 2 Department of Urology, Campus Lübeck, University Hospital Schleswig-Holstein, Lübeck, Germany; 3 Department of Surgery, S.H. Ho Urology Centre, The Chinese University of Hong Kong, Shatin, Hong Kong SAR China; 4 Hospital Israelita Albert Einstein, Sao Paulo, Brazil; 5 Department of Urology, Spital Thurgau AG (STGAG), Frauenfeld, Switzerland

**Keywords:** Chronic pelvic pain, Prostate, Transurethral surgery, Pain therapy, Real-world-data

## Abstract

**Background:**

Up to 50% of patients with benign prostate hyperplasia (BPH) face post-operative complications after transurethral prostate Surgery. This includes nearly 15% of patients suffering from prolonged post-operative pelvic pain (pPPP) consisting of dysuria and prostatodynia for sometimes several months post-surgery. This study elucidates and proposes a definition of the multi-dimensional nature of prolonged post-operative pelvic pain (pPPP) after transurethral prostate surgery by providing real-world data on therapeutic options and their efficacy.

**Methods:**

German and international urologic practitioners participated in an online-survey after invitation via social media accounts and newsletters. The survey included questions on the amount of expertise, the therapeutic regimens for prolonged post-operative pelvic pain (pPPP) and the expected therapy outcome. Participation was voluntary and uncompensated. Chi-Square tests and Student’s t-tests were used for descriptive statistics.

**Results:**

67 German urologists participated in a 9-question online Survey. 94,0% treated patients with lower urinary tract symptoms and 85,1% disclosed their therapeutic regime for prolonged post-operative pelvic pain (pPPP). The most common treatments included anti-inflammatory medication (69,6%), anti-cholinergics (53,6%), alpha-blockers (51,8%) and pelvic physiotherapy (50,0%). Over half of the patients responded to the therapeutic approach, but only 5,4% of urologists anticipated full pain relief after one year. These findings are closely aligned with a recent international survey (n=230). Notably, German urologists more frequently prescribed anti-cholinergics (53,6% vs. 28,7%, p = 0,0004), herbal remedies like saw palmetto (25,0% vs. 6,5%, p < 0,0001) and non-pharmacological therapies (82,1% vs. 49,1%, p < 0,0001), but less anti-inflammatory drugs (69,6% vs. 88,7%, p = 0,0003), gabapentin/pregabalin (8,9% vs. 42,2%, p < 0,0001) and opioids (0% vs 5,7%, p = 0,0221). Based on these results a structured definition and therapy plan for prolonged post-operative pelvic pain (pPPP) is proposed.

**Conclusion:**

Prolonged post-operative pelvic pain (pPPP) is a common challenge for urologists. Despite various therapeutic options, treatment outcomes and practitioner confidence remain suboptimal. Further research and attention to prolonged post-operative pelvic pain (pPPP) are essential to develop evidence-based guidelines for effective patient management and prevention of chronic pain syndromes.

**Supplementary Information:**

The online version contains supplementary material available at 10.1186/s12894-025-01943-z.

## Introduction

Benign prostatic hyperplasia (BPH) affects approximately 50% of men over 50 years, increasing to 90% in men over 90, making it one of the most prevalent conditions in aging males [1, 2]. Many affected individuals develop significant lower urinary tract symptoms (LUTS), necessitating therapeutic interventions including transurethral surgical approaches such as transurethral resection of the prostate (TUR-P) or holmium laser enucleation of the prostate (HoLEP) [3]. Post-operative complications, including persistent or de-novo LUTS, occur in 20–50% of patients following transurethral prostate surgery [4].

A recent review concluded that up to 15% of patients experience prolonged post-operative pelvic pain (pPPP) including dysuria (defined by The International Continence Society as “complaint of pain, burning or other discomfort during voiding”) and pelvic pain in men/prostatodynia (defined by The International Continence Society as “complaint of pain, pressure or discomfort related to the pelvis but not clearly related to the bladder, urethra, scortum or perineum”) for several months post-surgery, regardless of the surgical technique employed [5]. While often transient and self-limiting after three months[5], pPPP can significantly impair quality of life and may evolve from occasional discomfort into a chronic pain syndrome with multidimensional somatic, psychological, and social implications [6].

A recent international Survey of 230 urologists revealed that anti-inflammatory medications, alpha-blockers, and gabapentin/pregabalin are the most commonly prescribed treatments for pPPP [7]. Teoh et al. proposed a 6-step management approach emphasizing diagnosis (especially by ruling-out capsular perforation and urinary tract infection), therapy timing, and optimal treatment options, including pelvic physiotherapy and the aforementioned pharmaceutical options [7]. This study presents a similar online survey among German-speaking urologists, focusing on therapeutic strategies and expected outcomes for pPPP. The analysis and comparison of these two survey results should shed some light on the management of pPPP.

Currently, the condition of pPPP lacks a clear definition and structured diagnostic and therapeutic guidelines. Thus, this study aims to propose a conclusive clinical definition and practical therapy regimen of pPPP based on common clinical practice derived from the answers of experienced urologists in online surveys.

## Materials and methods

### German online survey

An online survey comprising nine questions in German was conducted using SurveyMonkey (SurveyMonkey Corporation, San Mateo, California, United States (US)) and distributed through the regular German Society of Urology (DGU) newsletter, ensuring broad representation within the German-speaking urological community. Participation was voluntary, anonymous and uncompensated. Table 1 presents the English translation of the questions, question types, and possible answers. The original German questionnaire is available in Supplementary Table 1.

**Table 1:** Questions of the German online survey in english translation

No.	Question	Possible Answers	Question Type
1	Did you execute diagnostics or therapy of LUTS on male adults within the last 24 months?	(1) Yes (2) No	Single response
2	Which age-group do you belong to?	(1) < 30 years (2) 30–49 years (3) 50–69 years (4) > 70 years	Single response
3	Which gender do you identify yourself?	(1) Female (2) Male (3) Other	Single response
4	Which term describes you current professional status best?	(1) Resident physician (2) Consultant (hospital) (3) Senior physician (4) Practitioner (5) None of the above mentioned	Single response
5	How do you treat patient with prolonged pelvic pain syndrome/prostatodynia after transurethral prostate surgery?	(1) Alpha-blockers (e.g. tamsulosine) (2) 5-alpha-reductase-blockers (e.g. finasteride) (3) Anti-cholinergic drugs (e.g. darifenacine, trospium chloride) (4) Antifungals (e.g. fluconazole) (5) Anti-inflammatory drugs (e.g. non-steroidal and anti-inflammatory drugs (NSAIDs), corticosteroids) (6) Antibiotics (7) Anticonvulsive drugs againt neuropathic pain (e.g. gabapentin, pregabalin) (8) Pelvic floor physiotherapy (9) Beta-3-agonists (e.g. mirabegrone) (10) Electro stimulation (e.g. transcutaneous electrical nerve stimulation (TENS)) (11) Muscle relexation (e.g. Baclofen) (12) Low-intensity extracorporal shockwave therapy (13) Opioids (14) Phenazopyridine (15) Phytotherapeutics (e.g. saw palmetto) (16) Physical therapy (e.g. sitz bath) (17) Serratiopeptidase (e.g. Emdase forte) (18) Other: free text	Multiple responses possible, free text
6	If you prescribe anti-inflammatory agents, which of the following do you prefer?	(1) NSAIDs suppository (2) Oral NSAIDs (3) Corticosteroids suppository (4) Oral corticosteroids (e.g. prednisone 5 mg/10 mg daily) (5) Corticosteroids s.c. (e.g. betamethasone 8 mg) (6) Corticosteroids i.v. (e.g. Dexamethason 8 mg) (7) I don’t prescribe anti-inflammatory drugs. (8) Other: free text	Multiple responses possible, free text
7	How long do you conduct the therapeutic option of choice until you consider it not effective?	(1) 2 weeks (2) 4 weeks (approximately 1 month) (3) 8 weeks (approximately 2 months) (4) 12 weeks (approximately 3 months) (5) 24 weeks (approximately 6 months) (6) Other: free text	Single response, free text
8	How many out of 10 patients with chronic pelvic pain syndrome/prostatodynia respond to the therapy?	(1) 0 (2) 1 (3) 2 (4) 3 (5) 4 (6) 5 (7) 6 (8) 7 (9) 8 (10) 9 (11) 10	Single response
9	How do you estimate your patients’ pain relief within one year compared to the start of therapy?	(1) No improvement (2) A little better (3) Half as much pain (4) Less than half as much pain (5) Scarcely pain at all (6) No complaints	Single response

### International online Survey [7]

A 5-question online survey was created using Google Form (Google LLC, Mountain View, California, US) and distributed via the #UroSeMe X platform (formerly: Twitter) in 2020 [8]. Participation was voluntary, anonymous and uncompensated. Table 2 displays the questions, question types, and possible answers.

**Table 2:** Questions of the international online survey[7]

No.	Question	Possible Answers	Question Type
1	Which best describes your current status?	(1) Urology Resident in Training (2) Urology Fellow (3) Urology Consultant	Single response
2	How would you treat patients with prolonged pelvic pain/prostatodynia after transurethral prostate surgery?	(1) Alpha-blocker (2) Anti-cholinergic (3) Anti-fungals (4) Anti-inflammatory agents (including NSAIDS, corticosteroids, etc.) (5) Antibiotics (6) Baclofen (7) Beta-3 agonist (8) Gabapentin/Pregabalin (9) Low-intensity extracorporeal shock wave therapy (10) Opioid (11) Pelvic physiotherapy (12) Phenazopyridine (13) Saw Palmetto (14) Serratiopeptidase (Emdase Forte) (15) Sitz bath (16) Others	Multiple responses possible
3	Which of the following anti-inflammatory medications do you prefer?	(1) NSAIDs suppository (2) Oral NSAIDs (3) Corticosteroids suppository (4) Oral corticosteroids (e.g. prednisone 5 mg/10 mg daily) (5) Intramuscular corticosteroids (e.g. betamethasone 8 mg) (6) Intravenous corticosteroids (e.g. dexamethasone 8 mg) (7) Others	Multiple responses possible
4	How long will you try the medication before you determine it is not effective?	(1) 2 weeks (2) 4 weeks (3) 8 weeks (4) 3 months (5) 6 months	Single response
5	Out of 10 patients with pelvic pain/prostatodynia, how many will respond to your treatment?	(1) 0 (2) 1 (3) 2 (4) 3 (5) 4 (6) 5 (7) 6 (8) 7 (9) 8 (10) 9 (11) 10	Single response

### Statistical analysis

Descriptive statistics were analysed using Microsoft Excel (Microsoft Corporation, Redmond, Washington, US). Comparisons between the two surveys were performed using Chi-Square tests and Student’s t-tests. P-values < 0.05 were considered statistically significant.

## Results

### German online survey

67 German-speaking urologic practitioners participated in the survey. Almost all participants (94.0%) treated LUTS patients within the last 24 months. Due to partial responses, the number of answers differs between the questions. 56.1% of participants were under 50 years and more than two-third identify themselves as male. More than 75% are board-certified urologists, while the others are residents. 42.1% of participants worked in private practice.

The majority prescribed anti-inflammatory drugs (69.6%), followed by anti-cholinergics (536%), alpha-blockers (51.8%) and pelvic physiotherapy (50.0%) in pPPP therapy. Furthermore, a substantial portion of participants recommended phytotherapeutics (25.0%) and sitz-bath (21.4%). Two participants included modern therapeutic modalities like digital mobile apps (especially Kranus Lutera [9]) and one urologist offered a multi-modal concept including psychotherapy. The importance of substantial diagnostics including cystoscopy and/or microbiological analysis was emphasized in free-text answers. Within the anti-inflammatory drugs, the respondents preferred oral NSAIDs (82.1%) over NSAID suppositories (21.4%) and oral corticosteroids (7.1%).

43,9% of participants considered four weeks adequate for therapy evaluation, while 17.5% only waited two weeks and 38.6% allowed eight weeks or more until considering the therapy as insufficient. The average expected therapy response was 5.53 out of ten patients. 5.4% of patients were considered to be pain-free and 48.9% to benefit by maximum 50% pain reduction after one year. Table 3 shows the detailed results of the German survey.

**Table 3:** Results of the German online survey

No.	Question	Answers	Count	Share
1	Did you execute diagnostics or therapy of LUTS on male adults within the last 24 months? (*n* = 67)	Yes	63	94.0%
		No	4	6.0%
2	Which age-group do you belong to? (*n* = 57)	< 30 years	4	7.0%
		30–49 years	28	49.1%
		50–69 years	25	43.9%
		> 70 years	0	0.0%
3	Which gender do you identify yourself? (*n* = 57)	Female	16	28.1%
		Male	39	68.4%
		Other	2	3.5%
4	Which term describes you current professional status best? (*n* = 57)	Resident physician	12	21.1%
		Fellow (hospital)	7	12.3%
		Senior physician	13	22.8%
		Fellow (practitioner)	24	42.1%
		None of the above mentioned	1	1.8%
5	How do you treat patient with prolonged pelvic pain syndrome/prostatodynia after transurethral prostate surgery? (*n* = 56)	Alpha-blockers (e.g. tamsulosine)	29	51.8%
		5-alpha-reductase-blockers (e.g. finasteride)	5	8.9%
		Anti-cholinergic drugs (e.g. darifenacine, trospium chloride)	30	53.6%
		Antifungals (e.g. fluconazole)	0	0.0%
		Anti-inflammatory drugs (e.g. non-steroidal and anti-inflammatory drugs (NSAIDs), corticosteroids)	39	69.6%
		Antibiotics	20	35.7%
		Anticonvulsive drugs againt neuropathic pain (e.g. gabapentin, pregabalin)	5	8.9%
		Pelvic floor physiotherapy	28	50.0%
		Beta-3-agonists (e.g. mirabegrone)	10	17.9%
		Electro stimulation (e.g. TENS)	4	7.1%
		Muscle relexation (e.g. Baclofen)	0	0.0%
		Low-intensity extracorporal shockwave therapy	2	3.6%
		Opioids	0	0.0%
		Phenazopyridine	1	1.8%
		Phytotherapeutics (e.g. saw palmetto)	14	25.0%
		Physical therapy (e.g. sitz bath)	12	21.4%
		Serratiopeptidase (e.g. Emdase forte)	0	0.0%
		Other	8	14.3%
6	If you prescribe anti-inflammatory agents, which of the following do you prefer? (*n* = 56)	NSAIDs suppository	12	21.4%
		Oral NSAIDs	46	82.1%
		Corticosteroids suppository	0	0%
		Oral corticosteroids (e.g. prednisone 5 mg/10 mg daily)	4	7.1%
		Corticosteroids s.c. (e.g. betamethasone 8 mg)	1	1.8%
		Corticosteroids i.v. (e.g. Dexamethason 8 mg)	0	0.0%
		I don’t prescribe anti-inflammatory drugs.	4	7.1%
		Other	1 (diclofenac)	1.8%
7	How long do conduct the therapeutic option of choice until you consider it no effective? (*n* = 57)	2 weeks	10	17.5%
		4 weeks (approximately 1 month)	25	43.9%
		8 weeks (approximately 2 months)	9	15.8%
		12 weeks (approximately 3 months)	13	22.8%
		24 weeks (approximately 6 months)	0	0.0%
		Other	2 (shorter)	3.5%
8	How many out of 10 patients with chronic pelvic pain syndrome/prostatodynia respond to the therapy? (*n* = 57)	0	1	1.8%
		1	0	0.0%
		2	2	3.5%
		3	6	10.5%
		4	8	14.0%
		5	15	26.3%
		6	4	7.0%
		7	9	15,8%
		8	10	17,5%
		9	1	1,8%
		10	1	1,8%
		Average:	5,53	
9	How do you estimate your patients’ pain relief within one year compared to the start of therapy? (*n* = 56)	No improvement	0	0,0%
		A little better	12	21,4%
		Half as much pain	21	37,5%
		Less than half as much pain	8	14,3%
		Scarcely pain at all	12	21,4%
		No complaints	3	5,4%

### International online survey [7]

230 international urologists participated in the online Survey from 2020, of whom 80% were urology consultants, 9.6% urology fellows and 10.4% were urology residents. The majority (88.7%) prescribed anti-inflammatory drugs in pPPP therapy, followed by alpha-blockers (42.2%), gabapentin/pregabalin (40.4) and pelvic physiotherapy (39.6%). If anti-inflammatory drugs were used, oral NSAIDs (81.3%) were preferred over NSAIDs suppositories (17.0%) and oral corticosteroids (17.0%). 48.3% of the respondents accepted four weeks until therapy evaluation, while 22.2% only waited two weeks. Around 30% of participants allowed the therapy regimen to run at least eight weeks. Only 0.9% of the participants expected all of their patients to respond to therapy, while 39.6% assumed that less than half of their patients will respond. The average expected therapy response was 5.9 out of ten patients. Table 4 shows the detailed results of the international survey.

**Table 4:** Results of the international online survey [7]

No.1	Question	Answers	Count	Share
1	Which best describes your current status? (*n* = 230)	Urology Resident in Training	24	10.4%
		Urology Fellow	22	9.6%
		Urology Consultant	184	80%
2	How would you treat patients with prolonged pelvic pain/prostatodynia after transurethral prostate surgery? (*n* = 230)	Alpha-blocker	97	42.2%
		Anti-cholinergic	66	28.7%
		Anti-fungals	2	0.9%
		Anti-inflammatory agents (including NSAIDS, corticosteroids, etc.)	204	88.7%
		Antibiotics	64	27.8%
		Baclofen	13	5.7%
		Beta-3 agonist	23	10.4%
		Gabapentin/Pregabalin	93	40.4%
		Low-intensity extracorporeal shock wave therapy	7	3.0%
		Opioid	20	8.7%
		Pelvic physiotherapy	91	39.6%
		Phenazopyridine	25	10.9%
		Saw Palmetto	15	6.5%
		Serratiopeptidase (Emdase Forte)	4	1.7%
		Sitz bath	15	6.5%
		Others	1 (Tadalafil)	0.4%
3	Which of the following anti-inflammatory medications do you prefer? (*n* = 230)	NSAIDs suppository	39	17.0%
		Oral NSAIDs	187	81.3%
		Corticosteroids suppository	9	3.9%
		Oral corticosteroids (e.g. prednisone 5 mg/10 mg daily)	39	17.0%
		Intramuscular corticosteroids (e.g. betamethasone 8 mg)	19	83%
		Intravenous corticosteroids (e.g. dexamethasone 8 mg)	4	1.7%
		Others	0	0.0%
4	How long will you try the medication before you determine it is not effective? (*n* = 230)	2 weeks	51	22.2%
		4 weeks	111	48.3%
		8 weeks	40	17.4%
		3 months	24	10.4%
		6 months	4	1.7%
5	Out of 10 patients with pelvic pain/prostatodynia, how many will respond to your treatment? (*n* = 230)	0	0	0.0%
		1	1	0.4%
		2	6	2.6%
		3	13	5.7%
		4	17	7.4%
		5	54	23.5%
		6	58	25.2%
		7	43	18.7%
		8	23	10.0%
		9	13	5.7%
		10	2	0.9%
		Average:	5,91	

### Comparison

A comparison between the German and international surveys revealed several significant differences in therapeutic approaches and demographics with a higher proportion of urology residents in the German survey (21.1% vs. 10.4%, *p* = 0.0303).

**Fig. 1:**
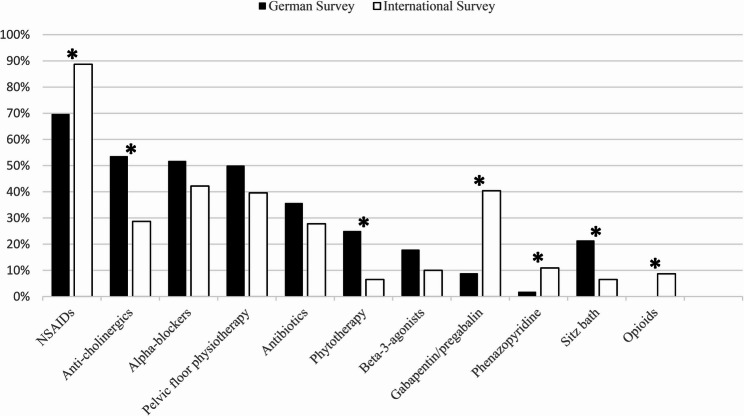
Comparison of treatment strategies indicating the percentage of participants that used the respective therapy option. *indicates statistical significance (*p* < 0.05)

Notably, a greater percentage of international respondents prescribed anti-inflammatory drugs (88.7% vs. 69.6%, *p* = 0.0003), gabapentin/pregabalin (42.2% vs. 8.9%, *p* < 0.0001), and opioids (5.7% vs. 0%, *p* = 0.0221). Conversely, German urologists showed a preference for anti-cholinergic medications (53.6% vs. 28.7%, *p* = 0.0004), herbal treatments like saw palmetto (25.0% vs. 6.5%, *p* < 0.0001), and non-pharmaceutical therapies (pelvic physiotherapy, sitz bath, low-intensity extra corporal shockwave therapy and electro stimulation: 82.1% vs. 49.1%, *p* < 0.0001). International respondents seem to also focus more often on the muscular aspect of pPPP and prescribed muscle-relaxing agents like baclofen (5.7% vs. 0.0%, *p* = 0.0686). These differences in treatment preferences between German and international urologists may reflect variations in clinical training, healthcare system structures, regulatory environments, and cultural attitudes towards pain management. The higher use of anti-inflammatory drugs, gabapentin/pregabalin, and opioids among international respondents may be influenced by broader prescribing practices and greater acceptance of pharmacological interventions for chronic pain in some countries. In contrast, the more conservative approach observed among German urologists - with a greater reliance on anticholinergic medications, phytotherapy (e.g., saw palmetto), and non-pharmaceutical therapies - may stem from a stronger emphasis on multimodal treatment strategies. Additionally, stricter regulations surrounding opioid prescription in Germany and greater caution regarding their long-term use may further explain the absence of opioid use among German respondents. Phenazopyridine is not approved in Germany, which explains its less frequent utilization compared to the results from the international survey (1.8% vs. 10.9%, *p* = 0.0340). Figure 1 shows the comparison of treatment strategies. Both groups favored oral NSAIDs over other forms (81.3% vs. 82.1%, *p* = 0.8849); however, German practitioners were more restrictive in their use of corticosteroids compared to their international counterparts (8.9% vs. 30.9%, *p* = 0.0009). This might be due to a higher emphasize on the long-term side effects of corticosteroids in the German speaking group.

The time frame for considering a therapy ineffective was similar between both groups, with international respondents favoring a maximum duration of two months for evaluation (87.3% vs. 77.2%, *p* = 0.0400). The expected response rates did not differ significantly between groups, with averages of 5.91 out of ten patients for international respondents compared to 5.53 out of ten for German respondents (*p* = 0.1869). All results are presented in Supplementary Table 2.

## Discussion

Despite new developments in transurethral prostate Surgery, a Substantial portion of patients of 20–50% is left with bothering symptoms of dysuria, urgency, infections or pain after surgery [4, 5, 10]. While some cases have identifiable causes like infections or perforations, the pathophysiology remains unclear for many patients [5]. Pathophysiologic theories include chronic bladder damage from long-term bladder-outlet-obstruction with collagenisation [11, 12] and damage caused by the heat of energy sources like laser and/or electricity during the surgical procedure [13]. Different studies reporting the outcome of various surgical approaches, including new minimally invasive procedures such as Aquablation, Urethral Lift and Rezum [14, 15], suggest a potential influence of the surgical approach to the nature and the rate of postoperative pelvic pain. Heat free methods like Urethral lift and Aquablation might prevent pain due to tissue damage caused by the heat, which might not be noticeable intraoperatively. Patients should be informed preoperatively about the advantages and disadvantages of the respective surgical approach and an individual decision should be made when selecting the treatment strategy with the patients, potentially including e.g. ultrasound parameters to predict the potential surgical outcome [16]. A recent meta-analysis on postoperative pelvic pain one month post-surgery Suggests a disadvantage for ablation procedures showing a rate of postoperative pain of 9% for enucleation, 10% for TURP but 15% for prostatic ablation [14]. Minimally invasive or heat free methods were, however, not included in the analysis. Furthermore, no randomized clinical trials are available, investigating postoperative pain for different surgical approaches when it comes to transurethral prostate surgery and while data suggests that clinical outcome is in part related to the occurrence of residual prostatic tissue [17], further studies are necessary to investigate the influence of residual tissue on the postoperative pain. Additionally, the Post-Voided Residual Ratio (PVR-R) - which measures the proportion of urine left in the bladder after voiding relative to the total bladder volume - was recently studied and rejected as a potential preoperative predictor for favorable outcomes after TURP [18], but evidence on its influence on postoperative pain is still missing.

Building on our current findings, it is important to address the current lack of a clear definition for pPPP. To fill this gap, we propose a clinical definition of pPPP grounded in our survey data, based on the following three key criteria:

a Complex of symptoms: pelvic and/or perineal pain, dysuria and pollakisuria;
b Timeframe after transurethral prostate surgery and duration: burden of symptoms for at least two consecutive weeks minimum two weeks post-surgery;
c Absence of post-operative complications (especially perforation or sphincter injury) and/or urinary tract infection (i.e. no bacteriuria).

This study with two online surveys among urologists shows that urologists frequently encounter pPPP patients based on the aforementioned definition. The therapeutic strategies mainly include symptomatic therapy focusing on pain and inflammation (including anti-inflammatory drugs and/or opioids) and addressing bladder emptying issues (alpha-blockers and/or anti-cholinergics), but also include non-pharmacological options (pelvic physiotherapy and sitz bath) or even multi-modal approaches aligned with the bio-psycho-social pain model [19]. Additionally, antibiotics are frequently used, when a postoperative infection is suspected. A recent study, investigating the role of the urinary microbiome in benign, non-infectious urological conditions [20], suggests that the microbiome is associated with symptom prevalence and severity of chronic pelvic pain syndrome, suggesting a potential influence on pain. Specifically, in chronic pelvic pain syndrome (CPPS), certain microbial taxa have been identified as linked to the condition. Some studies report reduced microbial diversity in patients with CPPS, indicating possible dysbiosis. Although no data currently exist on the postoperative microbiome and its impact on postoperative pain, the findings from this analysis strongly suggest a potential influence that warrants further investigation. Understanding this relationship could also reshape our approach to antibiotic use, as indiscriminate application may further disrupt the microbiome.

These findings align with existing literature on the use of NSAIDs [21, 22], oral corticosteroids [23, 24] and gabapentin [25] for pelvic pain management. Additional therapies such as wrist-ankle acupuncture [26], phosphodiesterase type 5 inhibitor [27] and transperineal botulinum toxin injection [28] have shown promise in early postoperative stage to help patients with pain or catheter-discomfort after transurethral prostate surgery. Systematic reviews support acupuncture and extracorporeal shockwave therapy [29], but find limited evidence for pharmacological interventions like anti-inflammatories, alpha-blockers or phytotherapy with a risk of side effects [30]. This structure is also followed in the European Guideline on chronic pelvic pain [31].

Fortunately, pPPP is self-limiting for a majority of cases, but some men develop a prolonged or chronic pain syndrome similar to chronic pelvic pain syndrome. This condition often includes psychosocial, emotional and sexual dysfunction [6, 32]. Latest at this point it is necessary to evaluate multi-disciplinary and multi-dimensional therapy options including psychotherapy [6, 31, 32].

No matter, how long the symptoms bother the patients; all of these patients need effective and evidence-based therapy. Therefore, we propose a sequential therapy concept for men reporting of bothersome symptoms including pain and dysuria after transurethral prostate surgery based on the aforementioned definition of pPPP:

1. Rule out post-operative complications like capsular/bladder perforation by ultrasound (and cystoscopy if needed).
2. Rule out urinary tract infection by urine dipstick and urine microbiology. Present bacteriuria (even if asymptomatic) should be treated with targeted antibiotics.
3. Offer symptomatic treatment from the beginning including pharmacological pain therapy (primarily NSAID and/or herbal therapeutics) and non-pharmacological therapy (pelvic physiotherapy, extracorporeal shockwave therapy and/or acupuncture).
4. Consider modifying the therapeutic regimen without symptomatic improvement after two weeks in a sequential manner. Explore second-line options like alpha-blockers, anti-cholinergics, corticosteroids or gabapentin/pregabalin, but keep in mind the potential side effects.
5. Consider pain specialist or psychologist consultation for multi-modal therapy after three months without improvement.

This easy-to-follow sequential therapy plan can sensitize urologists for pPPP and help patients to receive individual and effective therapy as soon as possible to minimize morbidity and limitation of quality of life. This might have a positive impact not only on the patient’s individual health but also for the healthcare system by reducing multiple hospital stays or visits of different practitioners and the economy by bringing the patients back to work earlier.

### Comparison to EAU guidelines

As outlined above, most urologists manage pPPP in alignment with the structure and principles of the EAU guidelines on chronic pelvic pain syndrome. Our suggested approach in this manuscript also aligns with these guidelines, while being specifically designed for men with persistent pain after transurethral prostate surgery. Therefore, a stronger emphasis is put on typical postoperative complications and proactive antibiotic treatment even for asymptomatic bacteriuria. Additionally, we propose a shorter, two-week window before escalating therapy, given the higher likelihood of a clinically relevant infection in the immediate postoperative period. In contrast to the EAU guidelines, we recommend postponing early multidisciplinary interventions - such as psychological support - since, in this context, the patient’s symptoms are more likely to be directly related to the surgical procedure rather than to psychosomatic or chronic pain mechanisms.

### Limitations of this study

Due to the small sample size, the results cannot be considered representative of the broader urologist population. However, the data nonetheless provide valuable preliminary insights. These findings can serve as a foundation for future larger-scale studies and help guide initial clinical considerations and hypothesis generation in this emerging area of research. Also, the findings are based on physicians’ perceptions and self-reported practices, which may not necessarily reflect real-world clinical outcomes or patient experiences. While survey-based studies offer valuable insights into current trends and expert opinions, they are inherently subjective and may be influenced by individual biases. Furthermore, using an online survey as the primary method of data collection may introduce a selection bias, as it is more likely to attract responses from younger, more digitally engaged urologists. This could potentially lead to an underrepresentation of older or less tech-savvy professionals, thereby influencing the generalizability of the result. However, by choosing the newsletter distributed by the German Association of Urology (DGU) for promoting this survey, we believe that our approach effectively targeted a broad and representative audience. The questionnaires lacked a clear definition of pPPP, which may have led participants to consider a variety of conditions. Additionally, the questionnaire used in this study is not a validated or standardized assessment tool. It was identical to a previously conducted survey, with the sole modification being its translation. This approach was chosen to enable a direct comparison with the results of the earlier published English-language survey. While this does not imply formal validation of the questionnaire, it does demonstrate that the tool has been successfully employed to collect relevant data in prior research.

## Conclusion

Prolonged post-operative pelvic pain (pPPP) is a common challenge for urologists, yet it remains a complex condition to treat effectively. Despite the availability of various therapeutic options, both the evidence base for successful outcomes and clinician confidence in treatment efficacy remain low. This study highlights the need for increased research focus and clinical attention on pPPP to improve patient care.

Moreover, there is no clear definition based on high-level evidence. Our proposed definition and consensus-based therapy plan, derived from comprehensive survey data of common clinical practice, aim to provide a foundation for standardized management and future research. By offering a structured approach to pPPP diagnosis and treatment, we hope to enhance clinical outcomes, facilitate earlier interventions, and ultimately improve quality of life for affected patients. This work serves as a stepping stone towards developing evidence-based guidelines and fostering further investigation into this challenging post-surgical complication.

## Supplementary Information

Supplementary Material 1.

## Data Availability

No datasets were generated or analysed during the current study.

## Abbreviations

BPH: Benign prostate hyperplasia
CPPS: Chronic pelvic pain syndrome
DGU: German Society of Urology
E.g.: For example
Et al.: Et alii
Etc.: Et cetera
HoLEP: Holmium laser enucleation of the prostate
I.v.: Intravenous
LUTS: Lower urinary tract symptoms
Mg: Milligram
NSAIDs: Non-steroidal and anti-inflammatory drugs
pPPP: Prolonged post-operative pelvic pain
S.c.: Subcutaneous
TENS: Transcutaneous electrical nerve stimulation
TUR-P: Transurethral resection of the prostate
US: United States
Vs.: Versus

## References

[1] Berry SJ, Coffey DS, Walsh PC, Ewing LL. The development of human benign prostatic hyperplasia with age. J Urol. 1984;132(3):474–9. 6206240 10.1016/s0022-5347(17)49698-4

[2] Launer BM, McVary KT, Ricke WA, Lloyd GL. The rising worldwide impact of benign prostatic hyperplasia. BJU Int. 2021;127(6):722–8. 33124118 10.1111/bju.15286 PMC8170717

[3] Uhlig A, Baunacke M, Groeben C, Borkowetz A, Volkmer B, Ahyai SA, et al. Die operative Therapie des benignen Prostatasyndroms in Deutschland. Urologe A. 2022;61(5):508–17. 35174398 10.1007/s00120-022-01777-9 PMC9072522

[4] Kim SJ, Al Hussein Alawamlh O, Chughtai B, Lee RK. Lower urinary tract symptoms following transurethral resection of prostate. Curr Urol Rep. 2018;19(10):85. 30128964 10.1007/s11934-018-0838-4

[5] Wroclawski ML, Castellani D, Heldwein FL, Teles SB, Cha JD, Zhao H, et al. Shedding light on polypragmasy of pain after transurethral prostate surgery procedures: a systematic review and meta-analysis. World J Urol. 2021;39(10):3711–20. 33787985 10.1007/s00345-021-03678-6

[6] Franz J, Kieselbach K, Lahmann C, Gratzke C, Miernik A. Chronic primary pelvic pain syndrome in men. Dtsch Arztebl Int. 2023;120(29–30):508–18. 36922749 10.3238/arztebl.m2023.0036 PMC10511008

[7] Teoh JYC, Wroclawski ML, Yuen S, Cheng B, Bertolo R, Castellani D, et al. Re: shedding light on polypragmasy of pain after transurethral prostate surgery procedures: a systematic review and meta-analysis. World J Urol. 2022;40(4):1057–9. 34021778 10.1007/s00345-021-03663-z PMC8994712

[8] Gudaru K, Blanco LT, Castellani D, Santamaria HT, Pelayo-Nieto M, Linden-Castro E, et al. Connecting the urological community: the #urosome experience. J Endoluminal Endourology. 2019;2(2):e21–9.

[9] Schönburg S, Gratzke C, Miller K, Wiemer L, Kliesch S. Digitale Gesundheitsanwendungen in der Urologie. Urologie. 2024;63(9):850–9. 39133296 10.1007/s00120-024-02398-0

[10] Ahyai SA, Gilling P, Kaplan SA, Kuntz RM, Madersbacher S, Montorsi F, et al. Meta-analysis of functional outcomes and complications following transurethral procedures for lower urinary tract symptoms resulting from benign prostatic enlargement. Eur Urol. 2010;58(3):384–97. 20825758 10.1016/j.eururo.2010.06.005

[11] Elbadawi A, Yalla SV, Resnick NM. Structural basis of geriatric voiding dysfunction. IV. Bladder outlet obstruction. J Urol. 1993;150(5 Pt 2):1681–95. 8411456 10.1016/s0022-5347(17)35869-x

[12] Su S, Lin J, Liang L, Liu L, Chen Z, Gao Y. The efficacy and safety of mirabegron on overactive bladder induced by benign prostatic hyperplasia in men receiving Tamsulosin therapy: A systematic review and meta-analysis. Med (Baltim). 2020;99(4):e18802. 10.1097/MD.0000000000018802 PMC7004736 31977871

[13] Castellani D, Pirola GM, Pacchetti A, Saredi G, Dellabella M. State of the art of thulium laser enucleation and vapoenucleation of the prostate: a systematic review. Urology. 2020;136:19–34. 31726185 10.1016/j.urology.2019.10.022

[14] Nguyen DD, Li T, Ferreira R, Baker Berjaoui M, Nguyen ALV, Chughtai B, et al. Ablative minimally invasive surgical therapies for benign prostatic hyperplasia: a review of aquablation, rezum, and transperineal laser prostate ablation. Prostate Cancer Prostatic Dis. 2024;27(1):22–8. 37081044 10.1038/s41391-023-00669-z

[15] Lombardo R, Santarelli V, Turchi B, Santoro G, Guercio A, Franco A, et al. Evaluation of peri-operative outcomes after prostatic urethral lift with emphasis on urodynamic changes, symptom improvement and sexual function. Diagnostics. 2024;14(19):2110. 39410516 10.3390/diagnostics14192110 PMC11475309

[16] DE Nunzio C, Voglino O, Cicione A, Tema G, Cindolo L, Bada M, et al. Ultrasound prostate parameters as predictors of successful trial without catheter after acute urinary retention in patients ongoing medical treatment for benign prostatic hyperplasia: a prospective multicenter study. Minerva Urol Nephrol. 2021;73(5):625–30. 33200904 10.23736/S2724-6051.20.04088-6

[17] Pyrgidis N, Mykoniatis I, Lusuardi L, Schulz GB, Sokolakis I, Stief C, et al. Enucleation of the prostate as retreatment for recurrent or residual benign prostatic obstruction: a systematic review and a meta-analysis. Prostate Cancer Prostatic Dis. 2023;26(4):693–701. 37193777 10.1038/s41391-023-00677-z

[18] Lombardo R, Ghezzo N, Sarcinelli L, Turchi B, Zammitti F, Franco A, et al. Post-voided residual ratio does not predict trifecta outcome after transurethral resection of prostate. Life. 2024;14(4):445. 38672716 10.3390/life14040445 PMC11051523

[19] Engel GL. The need for a new medical model: a challenge for biomedicine. Science. 1977;196(4286):129–36. 847460 10.1126/science.847460

[20] Suarez Arbelaez MC, Monshine J, Porto JG, Shah K, Singh PK, Roy S, et al. The emerging role of the urinary microbiome in benign noninfectious urological conditions: an up-to-date systematic review. World J Urol. 2023;41(11):2933–48. 37737900 10.1007/s00345-023-04588-5

[21] Kara C, Resorlu B, Cicekbilek I, Unsal A. Analgesic efficacy and safety of nonsteroidal anti-inflammatory drugs after transurethral resection of prostate. Int Braz J Urol. 2010;36(1):49–54. 20202235 10.1590/s1677-55382010000100008

[22] Donahue RP, Stamm AW, Daily AM, Kozlowski PM, Porter CR, Govier FE, et al. Opioid-Limiting pain control after transurethral resection of the prostate: A randomized controlled trial. Urology. 2022;166:202–8. 35314185 10.1016/j.urology.2022.03.010

[23] Bates S, Talbot M. Short course oral prednisolone therapy in chronic abacterial prostatitis and prostatodynia: case reports of three responders and one non-responder. Sex Transm Infect. 2000;76(5):398–9. 11141861 10.1136/sti.76.5.398 PMC1744215

[24] Jeong HJ. Effects of a short course of oral prednisolone in patients with bladder pain syndrome with fluctuating, worsening pain despite Low-Dose triple therapy. Int Neurourol J. 2012;16(4):175–80. 23346483 10.5213/inj.2012.16.4.175 PMC3547178

[25] Agarwal A, Dhiraaj S, Pawar S, Kapoor R, Gupta D, Singh PK. An evaluation of the efficacy of Gabapentin for prevention of catheter-related bladder discomfort: a prospective, randomized, placebo-controlled, double-blind study. Anesth Analg. 2007;105(5):1454–7. table of contents. 17959982 10.1213/01.ane.0000281154.03887.2b

[26] Hou J, Li Y, Wu Y, Liu Y, Chen Q, Li Y, et al. Safety and efficacy of wrist-ankle acupuncture in treating catheter-related bladder discomfort after transurethral resection of the prostate: a double-blind randomized clinical trial. Gland Surg. 2022;11(9):1464–71. 36221271 10.21037/gs-22-438 PMC9547715

[27] Kirby RS, Carson C, Dasgupta P. Daily phosphodiesterase type 5 inhibitor therapy: a new treatment option for prostatitis/prostatodynia? BJU Int. 2014;113(5):694–5. 24877211 10.1111/bju.12681

[28] Li C, Ji F, Fan F, Xu J, Xu H. Transperineal botulinum toxin injection for chronic pelvic pain syndrome after transurethral resection of the prostate. Urol J. 2022;19(4):333–8. 35762081 10.22037/uj.v19i.7128

[29] Franco JVA, Turk T, Jung JH, Xiao YT, Iakhno S, Garrote V, et al. Non-pharmacological interventions for treating chronic prostatitis/chronic pelvic pain syndrome: a Cochrane systematic review. BJU Int. 2019;124(2):197–208. 30019814 10.1111/bju.14492

[30] Franco JVA, Turk T, Jung JH, Xiao YT, Iakhno S, Tirapegui FI, et al. Pharmacological interventions for treating chronic prostatitis/chronic pelvic pain syndrome: a cochrane systematic review. BJU Int. 2020;125(4):490–6. 31899937 10.1111/bju.14988

[31] Engeler D. EAU guidelines on chronic pelvic pain. European Association of Urology; 2025. 10.1016/j.eururo.2009.08.020 19733958

[32] Brünahl CA, Klotz SGR, Dybowski C, Albrecht R, Höink J, Fisch M, et al. Physiotherapy and combined cognitive-behavioural therapy for patients with chronic pelvic pain syndrome: results of a non-randomised controlled feasibility trial. BMJ Open. 2021;11(12):e053421. 34907064 10.1136/bmjopen-2021-053421 PMC8671982

