# Supplementary material for: Management of prolonged post-operative pelvic pain after transurethral prostate surgery: a clinical real-world survey and international comparison of therapy regimens
Source: BMC Urol. 2025 Sep 17;25:231. doi: 10.1186/s12894-025-01943-z (PMC12442294; doi:10.1186/s12894-025-01943-z)
Supplement: Supplementary file 1 — Supplementary Material 1. [file 12894_2025_1943_MOESM1_ESM.docx]

**Supplementary Material**

| No. | Question | Possible Answers |
| --- | --- | --- |
| 1 | Haben Sie Diagnostik oder Behandlungen von LUTS in den letzten 24 Monaten an männlichen Erwachsenen durchgeführt? | 1. Ja 2. Nein |
| 2 | In welche Altersgruppe können Sie sich zuordnen? | 1. <30 Jahre 2. 30-49 Jahre 3. 50-69 Jahre 4. >70 Jahre |
| 3 | Welchem Geschlecht möchten Sie sich zuordnen? | 1. Weiblich 2. Männlich 3. Weitere Geschlechtsform |
| 4 | Welcher Terminus würde Ihren aktuellen Berufsstand am ehesten beschreiben? | 1. Arzt/Ärztin in der Fortbildung zum Facharzt/-ärztin für Urologie (Assistenzarzt) 2. Facharzt/-ärztin für Urologie an einer Klinik 3. Oberarzt/-ärztin 4. Niedergelassener Facharzt/-ärztin für Urologie 5. Keine der oben genannten |
| 5 | Wie behandeln Sie üblicherweise Patienten mit prolongiertem Beckenschmerzsyndrom/ Prostatodynie nach einer transurethralen Prostata OP?  Mehrfachantworten sind möglich | 1. Alpha-Blocker (z.B. Tamsulosin) 2. 5-Alpha-Redukatse-Hemmer (z.B. Finasterid) 3. Anticholinergika (z.B. Darifenancin, Trospium chlorid) 4. Antimykotika (z.B. Fluconazol) 5. Antiinflammative Substanzen (z.B. NSAIDs, Kortikosteroide) 6. Antibiotika 7. Antikonvulsiva gegen enrupathische Schmerzen (z.B. Gabapentin, Pregabalin) 8. Beckenbodentraining 9. Beta-3—Agnositen (z.B. Mirabegron) 10. Elektrostimulation (z.B. TENS) 11. Muskelrelaxanzen (z.B. Baclofen) 12. Niedrigenergetische Extrakorporale Schockwellentherapie (ESWT) 13. Opioide 14. Phenazopyridine 15. Phytoptherapie (z.B. Sägepalmenextrakt) 16. Physikalische Therapie (z.B. Sitzbäder) 17. Serratiopeptidase (z.B. Emdase Forte) 18. Sonstiges: Textfeld |
| 6 | Falls Sie entzündungshemmende Substanzen verschreiben, welche der unten aufgeführten Medikamente bevorzugen Sie typischerweise?  Mehrfachantworten sind möglich. | 1. NSAIDs Zäpfchen 2. NSAIDs p.o. 3. Kortikosteroid Zäpfchen 4. Kortikosteroide p.o. (z.B. Prednisone 5mg/10 mg täglich) 5. Kortikosteroide s.c. (z.B. Betamethason 8 mg) 6. Kortikosteroide i.v. (z.B. Dexamethason 8 mg) 7. Ich verschreibe keine entzündungshemmenden Substanten 8. Sonstiges: Textfeld |
| 7 | Wie lange führen Sie eine Behandlungsoption durch, bis Sie diese als nicht wirksam bewerten? | 1. 2 Wochen 2. 4 Wochen (ca. 1 Monat) 3. 8 Wochen (ca. 2 Monaten) 4. 12 Wochen (ca. 3 Monate) 5. 24 Wochen (ca. 6 Monate) 6. Sonstiges: Textfeld |
| 8 | Von 10 behandelten Patienten mit chronischem Beckenschmerzsyndrom/ Prostatodynie, wie viele sprechen auf die Therapie an? | 1. 0 2. 1 3. 2 4. 3 5. 4 6. 5 7. 6 8. 7 9. 8 10. 9 11. 10 |
| 9 | Im Vergleich zu den Schmerzen bei Therapiebeginn, wie schätzen Ihre Patienten insgesamt Ihre Schmerzverbesserung innerhalb eines Jahres ein? | 1. Keine Verbesserung 2. Ein wenig besser 3. Halb so viele Schmerzen 4. Mehr als doppelt so gut 5. Kaum noch schmerzen 6. Beschwerdefrei |

Supplementary Table 1: Original questions and answers in German language.

| Question | Answers | German survey | International survey | Significance |
| --- | --- | --- | --- | --- |
| Which best describes your current status? | Resident | 21.1% | 10.4% | 0.0303* |
|  | Fellow | 35.1% | 9.6% | <0.0001* |
|  | Consultant | 42.1% | 80% | <0.0001* |
| How do you treat patient with prolonged pelvic pain syndrome / prostatodynia after transurethral prostate surgery? | Anti-inflammatory medication | 69.6% | 88.7% | 0.0003* |
|  | Alpha-Blocker | 51.8% | 42.2% | 0.1939 |
|  | Gabapentin / Pregabalin | 8.9% | 40.4% | <0.0001* |
|  | Pelvic physiotherapy | 50.0% | 39.6% | 0.1554 |
|  | Anti-cholinergic medication | 53.6% | 28.7% | 0.0004* |
|  | Antibiotics | 35.7% | 27.8% | 0.2451 |
|  | Phenazopyridine | 1.8% | 10.9% | 0.0340* |
|  | Beta-3 agonist | 17.9% | 10.0% | 0.0989 |
|  | Opioid | 0.0% | 8.7% | 0.0221* |
|  | Saw palmetto | 25.0% | 6.5% | <0.0001* |
|  | Sitz bath | 21.4% | 6.5% | 0.0006* |
|  | Baclofen | 0.0% | 5.7% | 0.0686 |
|  | Amitriptyline | n.a. | 3.0% | n.a. |
|  | Low-intensity ESWT | 3.6% | 3.0% | 0.8392 |
|  | Serratiopeptidase | 0.0% | 1.7% | 0.3203 |
|  | Anti-fungals | 0.0% | 0.9% | 0.4838 |
|  | 5-alpha-Reduktase-Hemmer | 8.9% | n.a. | n.a. |
|  | Electro Stimulation | 7.1% | n.a. | n.a. |
|  | Other | 14.3% | n.a. | n.a. |
|  | All non-medication | 82.1% | 49.1% | <0.0001* |
| Which of the following anti-inflammatory medications do you prefer? | Oral NSAIDs | 82.4% | 81.3% | 0.8849 |
|  | NSAID suppository | 21.4% | 17.0% | 0.4330 |
|  | Corticosteroids p.o. | 7.1% | 17.0% | 0.0654 |
|  | Corticosteroids i.m. | n.a. | 8.3% | n.a. |
|  | Corticosteroids suppository | 0.0% | 3.9% | 0.1325 |
|  | Cotricosteroids i.v. | 0.0% | 1.7% | 0.3203 |
|  | Corticosteroids s.c. | 1.8% | n.a. | n.a. |
|  | All corticosteroids | 8.9% | 30.9% | 0.0009* |
| How long will you try the medication before you determine it is not effective? | 2 weeks | 17.5% | 22.2% | 0.4443 |
|  | 4 weeks | 43.9% | 48.3% | 0.5513 |
|  | 8 weeks | 15.8% | 17.4% | 0.7736 |
|  | 3 months | 22.8% | 10.4% | 0.0126* |
|  | 6 months | 0.0% | 1.7% | 0.3160 |
|  | Maximum 2 months | 77.2% | 87.3% | 0.0400* |
| Out of 10 patients with pelvic pain / prostatodynia, how many will respond to your treatment? | 0 | 1.8% | 0.0% | 0.0442* |
|  | 1 | 0.0% | 0.4% | 0.6186 |
|  | 2 | 3.5% | 2.6% | 0.7117 |
|  | 3 | 10.5% | 5.7% | 0.1852 |
|  | 4 | 14.0% | 7.4% | 0.1113 |
|  | 5 | 26.3% | 23.5% | 0.6536 |
|  | 6 | 7.0% | 25.2% | 0.0028* |
|  | 7 | 15.8% | 18.7% | 0.6101 |
|  | 8 | 17.5% | 10.0% | 0.1100 |
|  | 9 | 1.8% | 5.7% | 0.2213 |
|  | 10 | 1.8% | 0.9% | 0.5565 |
|  | Average | 5.53 | 5.91 | 0.1869 |

Supplementary Table 2: Comparison between international and German participants. N.a. = not applicable. *stands for significance defined as p-value <0.05.
